# Regulatory focus and generalized trust: the impact of prevention-focused self-regulation on trusting others

**DOI:** 10.3389/fpsyg.2015.00254

**Published:** 2015-03-17

**Authors:** Johannes Keller, Ruth Mayo, Rainer Greifeneder, Stefan Pfattheicher

**Affiliations:** ^1^Abteilung Sozialpsychologie, Institut für Psychologie und Pӓdagogik, University of UlmUlm, Germany,; ^2^The Hebrew University of JerusalemJerusalem, Israel,; ^3^University of BaselBasel, Switzerland

**Keywords:** generalized trust/distrust, regulatory focus, self-regulation, sensitivity to negative information

## Abstract

The current research suggests that taking self-regulatory mechanisms into account provides insights regarding individuals’ responses to threats in social interactions. In general, based on the notion that a prevention-focused orientation of self-regulation is associated with a need for security and a vigilant tendency to avoid losses and other types of negative events we advocate that a prevention-focused orientation, both as a disposition as well as a situationally induced state, lowers generalized trust, thus hindering cooperation within social interactions that entail threats. Specifically, we found that the more individuals’ habitual self-regulatory orientation is dominated by a prevention focus, the less likely they are to score high on a self-report measure of generalized trust (Study 1), and to express trust in a trust game paradigm as manifested in lower sums of transferred money (Studies 2 and 3). Similar findings were found when prevention focus was situationally manipulated (Study 4). Finally, one possible factor underlying the impact of prevention-focused self-regulation on generalized trust was demonstrated as individuals with a special sensitivity to negative information were significantly affected by a subtle prevention focus manipulation (versus control condition) in that they reacted with reduced trust in the trust game (Study 5). In sum, the current findings document the crucial relevance of self-regulatory orientations as conceptualized in regulatory focus theory regarding generalized trust and responses to threats within a social interaction. The theoretical and applied implications of the findings are discussed.

## Introduction

### Vigilant Prevention-Focused Self-Regulation and Generalized Trust

Self-regulatory orientations influence many fundamental social cognitive as well as social interactive mechanisms and affect individuals’ thought processes, emotions, and behavioral tendencies in social interactions (for an overview of the field of self-regulation research, see the volume edited by Vohs and Baumeister, 2011). One theoretical perspective figures particularly prominently in this research on self-regulatory orientations: regulatory focus theory (RFT) introduced by Higgins (1997, 1998, 2012a,b). The present contribution aims to explore the role of RFT with respect to a fundamental and important social interactive phenomenon: generalized trust. Specifically, we address the relations between prevention- and promotion-focused self-regulation and generalized trust, mainly as affecting responses to social interactions that involve threats. The basal motivational orientations as conceptualized in RFT play a crucial role in dealing with threats and uncertainty (prevention focus) as well as with opportunities for growth and attainment of maximal goals (promotion focus). Situations where a decision is pending whether one is willing to trust others reflect a state of uncertainty in that one is exposed to the threat that trusting others may prove to be disadvantageous. In this regard, we propose that especially prevention-focused self-regulation is crucial regarding the willingness to trust others in social interactions that entail threats. Overall, the present research adds to and extends a growing body of research, which puts the spotlight on the self-regulatory character of psychological phenomena (cf. Carver, 2006).

To build a common basis for our arguments, we start out with a brief discussion of the core assumptions proposed in RFT. Based on this, we offer a conceptual analysis to explore the relation between prevention-focused self-regulation and generalized trust.

### Core Assumptions of Regulatory Focus Theory

Extending the basic hedonic principle that people approach pleasure and avoid pain, RFT holds that it is necessary to differentiate distinct types of pleasures and distinct types of pain and to assess the specific strategic orientations and types of goal pursuit that reflect self-regulation guided by two distinct motivational systems – promotion focus and prevention focus (Higgins, 1998, 2012a,b; Molden et al., 2008). Self-regulation with a promotion focus is characterized as the motivation to attain growth and nurturance, to bring one’s actual self into alignment with one’s ideal self, as well as the desire to reach gains (and to avoid non-gains). In contrast, self-regulation with a prevention focus entails the motivation to attain security, to bring one’s actual self into alignment with one’s ought self (i.e., fulfilling one’s duties and obligations), as well as the desire to avoid losses (and to attain non-losses).

Both types of regulatory orientations are presumed to be related to specific consequences. RFT postulates several consequences of self-regulation with a promotion focus: (a) a special sensitivity to the presence or absence of *positive* outcomes, (b) application of *eager* strategic means (i.e., to insure hits and to insure against errors of omission), (c) ambitious and keen striving to reach one’s aspirations as reflected in tenacious goal pursuit that is focused on *maximal* goals (i.e., goals differentiating a positive region of outcomes from a non-positive/neutral region; cf. Brendl and Higgins, 1996), and (d) *cheerfulness-dejection* emotions in response to positive and negative events. In contrast, according to RFT, self-regulation with a prevention focus is associated with the following consequences: (a) a special sensitivity to the presence or absence of *negative* outcomes, (b) application of *vigilant* strategic means (i.e., to insure correct rejections and to insure against errors of commission), (c) a defensive orientation in the pursuit of *minimal* goals (i.e., goals differentiating a negative region of outcomes from a non-negative/neutral region; cf. Brendl and Higgins, 1996), and (d) *quiescence-agitation* emotions in response to positive and negative events. These theoretical assumptions have been supported by substantial empirical evidence (see Higgins, 1998, 2012a,b; Higgins and Spiegel, 2004, for reviews).

Particularly important in the present context, RFT posits that individuals may differ in their predominant *chronic* or *habitual* self-regulatory orientation, and several measures to assess these individual differences have been developed (e.g., regulatory focus questionnaires, cf. Higgins et al., 2001; Lockwood et al., 2002; Keller, unpublished regulatory strength measures, cf. Shah et al., 1998). The two self-regulatory systems can also be manipulated, that is, situationally induced or primed (Roney et al., 1995; Shah et al., 1998; Friedman and Förster, 2001; Freitas et al., 2002).

It is important to note that the two modes of self-regulation, promotion and prevention, have been conceptualized as independent constructs and therefore may vary independently. That is, individuals can be high on both, low on both, or predominantly prevention- or promotion-focused. Accordingly, measures of the two modes of self-regulation (chronic promotion and prevention-focused orientation) have been found to be largely uncorrelated or slightly positively correlated (cf. Higgins et al., 2001; Lockwood et al., 2002; Keller, unpublished). Promotion-focused self-regulation is thus *not* the opposite pole of prevention-focused self-regulation. In consequence, the two modes of self-regulation may display very different patterns of relations with other constructs, such that one of the two modes may be related to a certain phenomenon or construct while the other mode is not.

The present contribution aims to explore the role of RFT with respect to the fundamental and important social interactive phenomenon of trust, specifically as reflected in response to social situations that entail threats. Based on the notion that a *prevention-focused* orientation of self-regulation is associated with a need for security and the desire to attain it, a tendency to avoid dangers and threats, as well as a proclivity to be defensive and vigilant, we hypothesized that such an orientation is likely to lower generalized trust, in turn hindering the potential for cooperation in a social interaction. Given that trust involves a willingness to accept vulnerability and to take risks – a tendency which appears largely incompatible with a concern for safety and security – it seems reasonable to assume a negative relation between a prevention-focused mode of self-regulation and trust. We discuss the general logic underlying this proposition in the next paragraphs.

### Trust and Its Relation to Self-Regulatory Mechanisms

According to the Oxford English Dictionary, trust is “[c]onfidence in or reliance on some quality or attribute of a person or thing, or the truth of a statement," and the act of trusting is defined as “to accept or give credit to without investigation or evidence." What is probably the most widely cited definition of trust in the psychological literature was introduced by Rotter (1971, p. 444) who defined trust as “an expectancy held by an individual or a group that the word, promise, verbal, or written statement of another individual or a group can be relied upon." In all of these definitions, trust is seen as lending credibility to a person or group without (conclusive) knowledge about the actual credibility or reliability of the person or group. This aspect also figures prominently in Rempel et al.’s (1985) conceptualization of trust, which refers to predictability, dependability (the willingness to put oneself at risk through reliance on another’s promises), and faith (confidence in caring responses) as crucial components. In a more recent definition, Rousseau et al. (1998, p. 395) proposed a conceptualization of trust according to which “[t]rust is a psychological state comprising the intention to accept vulnerability based upon positive expectations of the intentions or behavior of another.". This perspective specifically highlights an aspect of trust that is also (if only implicitly) entailed in the definitions mentioned above, namely that trust involves a certain level of *willingness to accept risks and uncertainty* (in the sense that the individual accepts the possibility that he or she might suffer a loss, injury, or harm as a consequence of engaging in an interaction) based on positive expectations about the other person’s intentions. Thus, the core of generalized trust is the global belief that people are likely to be reliable, sincere, cooperative, benevolent, and truthful (Simpson, 2007).

As mentioned, our work focuses on *generalized* trust. This perspective differs from the dyadic interpersonal perspective on trust that rose in the partnership- and relationship-centered programs of research initiated in the 1980s (cf. Larzelere and Huston, 1980; Johnson-George and Swap, 1982; Rempel et al., 1985; Holmes and Rempel, 1989). Specifically, trusting a well-known partner in a close relationship might be different to generalized trust including unknown others and people in general (cf. General Discussion).

Trust can be examined from several perspectives and levels. On the *cognitive* level, trust entails the expectation that most others have benign intentions (e.g., Acar-Burkay et al., 2014). In social interactions individuals often do not know how other individuals will behave. That is to say, many social interactions entail a threat that one is exploited by uncooperative and antisocial behavior of others (e.g., Balliet and Van Lange, 2013a,b). In threatening social situations trust decreases, especially among individuals who possess few resources and who feel powerless to deal with the threat (Ross, 2011). However, if individuals do not trust others, they miss the possibility of beneficial social interactions and positive reciprocity (Berg et al., 1995; McCabe et al., 2003). Some amount of trust is therefore necessary for ongoing and beneficial social interactions.

On the *behavioral* level, trust reflects a willingness to accept a state of dependency on another individual who has the power to return harm or benefits (Ainsworth et al., 2014). Behaving trustworthily toward others increases cooperative behavior in social groups (Balliet and Van Lange, 2013a) and lowers transaction costs because interaction partners need to be less monitored (Cook et al., 2005). In close relationships, showing trust is positively related to felt security, constructive strategies in coping, and emphasizing positive aspects of the relationship (Mikulincer, 1998; Rempel et al., 2001). Thus, showing trust also counters uncertain and threatening social situations.

Research regarding trust – and its counterpart, distrust – has also included the prevalence of deception in our life (DePaulo et al., 1996; DePaulo and Kashy, 1998; Feldman et al., 2002), and social perceivers’ attempts (usually unsuccessful) to decode deception (DePaulo et al., 1997; Anderson et al., 1999) and the differentiation of cooperators from deceivers (Yamagishi et al., 2003). Recent research has investigated the impact of hormones, especially oxytocin, on trust behavior (Kosfeld et al., 2005; Van IJzendoorn and Bakermans-Kranenburg, 2012). However, somewhat surprisingly, the role of basic *motivational* mechanisms with respect to generalized trust has been largely neglected to date. Given the eminent relevance of trust in social life, it seems important to understand the underlying motivational mechanisms involved in generalized trust, trust behavior, as well as the boundary conditions that may lead to (or inhibit) the expression of generalized trust. We address this issue by focusing specifically on the role of basic self-regulatory mechanisms as proposed in RFT.

As outlined in RFT (Higgins, 1997, 1998, 2012a,b), prevention-focused self-regulation reflects a desire to avoid losses and need for security, the desire to reach safety. In this regard, particularly prevention-focused individuals consider security to reflect a desirable human value (Leikas et al., 2009). Moreover, prevention-focused self-regulation is associated with a special sensitivity to (potential) negative events (e.g., Keller and Pfattheicher, 2013; Pfattheicher and Sassenrath, 2014). In this sense, Pfattheicher and Keller (2013) showed that strongly prevention-focused individuals punish antisocial others in a social dilemma situation more than weakly prevention-focused individuals. Neural correlates also supports this assumption indicating a greater activity in the amygdala, anterior cingulate, and extrastriate cortex for prevention-focused individuals when negative (versus positive) information is presented (Cunningham et al., 2005). Furthermore, prevention-focused self-regulation has been related to vigilant and careful strategic tendencies reflecting threat, defensiveness, and cautiousness (note that evidence documenting a significant negative correlation between measures of a prevention-focused self-regulatory orientation and sensation-seeking as a measure of disinhibition supports this notion; Uskul et al., 2008; cf. Keller, unpublished).

Social situations that demand trusting others entail a threat that one is exploited by uncooperative and antisocial behavior of others (e.g., Balliet and Van Lange, 2013a,b). That is to say, when trusting others individuals are in a situation of insecurity, vulnerability, and uncertainty. As is evident from this list of concepts that are related to the prevention focus, a prevention-focused self-regulatory orientation reflects a mode of self-regulation that is concerned with security while avoiding negative events to happen. In fact, the defining concepts that characterize trust and prevention focus seem to be incompatible (e.g., the willingness to accept vulnerability, uncertainty, and risk as characteristic feature of trust compared to a concern for safety and security in prevention focus). Accordingly, we assume that there is a negative relation between a prevention-focused mode of self-regulation and generalized trust.

In contrast to the close conceptual relation between prevention-focused self-regulation and trust, the defining aspects involved in the conceptualization of promotion-focused self-regulation appear not particularly strongly associated with the willingness to accept vulnerability, uncertainty, and risk, as the defining characteristic of trust. Moreover, since both types of self-regulation (promotion and prevention) have been conceptualized as independent constructs, proposing a negative association between prevention focus and generalized trust does not imply the reverse, that is to say, a positive association between promotion focus and generalized trust.

In sum, the conceptual analysis outlined above leads us to hypothesize a negative relation between prevention-focused self-regulation and generalized trust. In contrast, based on the conceptual analysis outlined above, it seems most plausible to expect that promotion-focused self-regulation is largely unrelated to generalized trust. As a starting point, we tested these basic assumptions in three studies assessing the relation between self-report measures of regulatory focus and (a) a self-report measure of generalized trust (Studies 1a,b), and (b) a measure designed to assess the behavioral tendency to trust another unfamiliar person based on the experimental trust game paradigm as a case of social interaction that entails a threat (Studies 2–5). To further bolster confidence in these findings, we manipulated prevention focus in Study 4. Finally, going beyond the global relationship between prevention-focused self-regulation and generalized trust, we elaborate on the specific mechanisms that may be involved by manipulating a prevention focus problem cue and testing its effect on trust behavior as a function of one specific dispositional tendency (sensitivity to negative information) that is known as a characteristic element of prevention-focused self-regulation (Study 5).

All studies of the present work were conducted in line with the ethical standards of the American Psychological Association (APA). All participants were given written informed consent prior to the study. In order to assure anonymity, participants did not provide information that allows inferences to the participants (e.g., names). Participants were paid in private at the end of each study and debriefed.

## Study 1

The goal of Study 1 was to assess whether individual differences in generalized trust are related to differences in chronic regulatory focus.

## Procedure

This study involved two independent samples of students at the University of Mannheim (Study 1a: *N* = 88; *M*_age_ = 22.9; 45 females; Study 1b: *N* = 117; *M*_age_ = 23.3; 56 females) ^1^. Participants in the two questionnaire studies completed measures designed to assess habitual levels of generalized trust and regulatory focus. In Study 1a, responses were assessed using 9-point response scales; in Study 1b, we used 7-point scales. In both studies, scale endpoints were labeled *not at all true* and *completely true*. We used a German version of Rotter’s (1967) trust scale to assess generalized trust (Amelang et al., 1984). A sample item of this measure reads: “In dealing with strangers one is better off being cautious until they have provided evidence that they are trustworthy." The scale reached Cronbach’s alpha levels of 0.76 (Study 1a) and 0.77 (Study 1b).

We assessed regulatory orientations using a German version (Keller and Bless, 2006) of the regulatory focus scale developed by Lockwood et al. (2002), which consists of prevention and promotion focus subscales. A prevention scale sample item reads: “In general, I am focused on preventing negative events in my life." And a promotion scale sample item reads: “I frequently imagine how I will achieve my hopes and aspirations." The promotion scale reached Cronbach’s alpha levels of 0.77 (Study 1a) and 0.81 (Study 1b); the prevention scale reached Cronbach’s alpha levels of 0.76 (Study 1a) and 0.80 (Study 1b) ^2^.

In Study 1b, additional measures of basic dimensions of personality (extraversion; neuroticism) were assessed using a German version (Eggert, 1983) of Eysenck’s personality inventory in order to test whether the assumed association between individuals’ regulatory orientation and their level of generalized trust remains robust when controlling for these broad personality dimensions. Both scales reached acceptable levels of internal validity, Cronbach’s α = 0.78 (extraversion) and 0.87 (neuroticism).

## Results and Discussion

**FIGURE 1:**
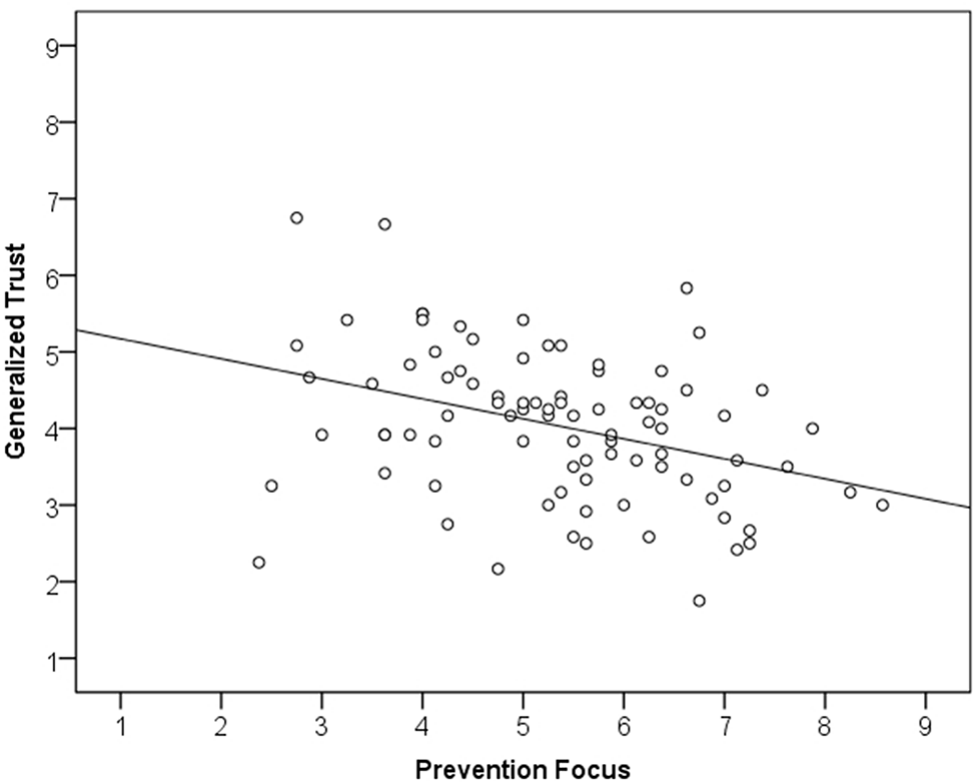
X–Y plot of Study 1a.

**FIGURE 2:**
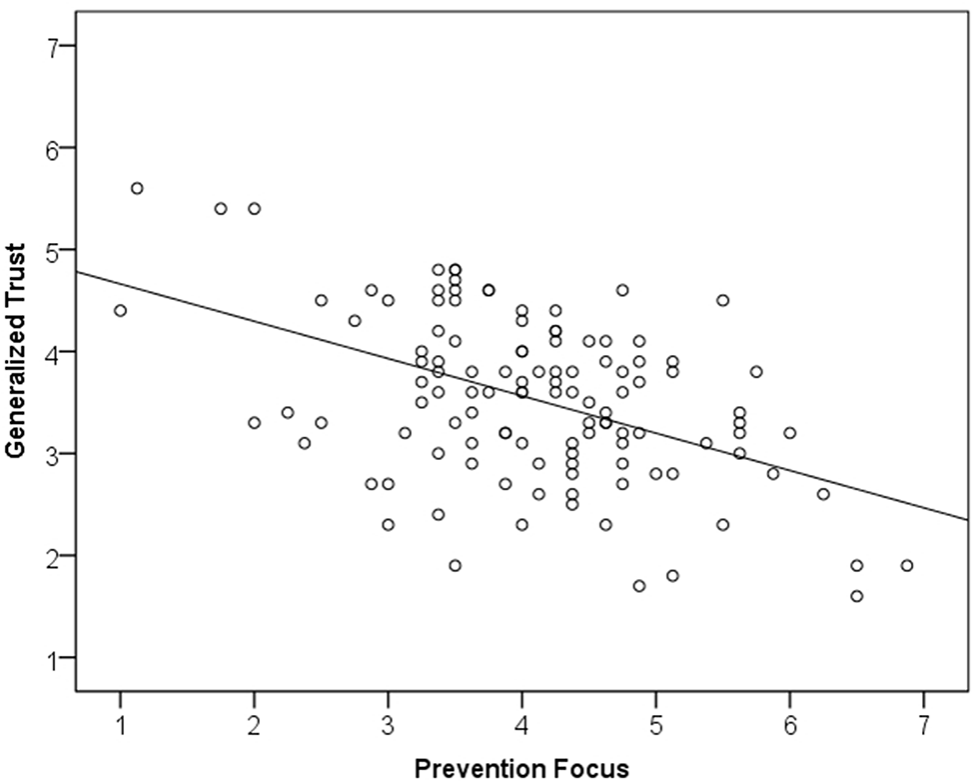
X–Y plot of Study 1b.

To test the relations between regulatory orientation and generalized trust we computed zero-order correlations (see **Table 1**) as well as regression analyses. In both samples (Studies 1a,b) prevention focus was significantly and negatively correlated with generalized trust (see Figures **1 and 2**). These results support the proposed negative association between the two constructs. The regression analyses controlling for promotion scores (Study 1a; top panel of **Table 2**) as well as scores on the neuroticism and extraversion scales (Study 1b; bottom panel of **Table 2**) reveal that this relation is robust.

**Table 1:** Zero-order correlations between variables included in Studies 1a,b.

	Prevention	Promotion	Generalized trust	Neuroticism	Extraversion
Prevention	–	-0.02	-0.47^∗∗∗^	0.69^∗∗∗^	-0.27^∗∗^
Promotion	0.22^∗^	–	-0.19^∗^	-0.08	0.28^∗∗^
Generalized trust	-0.37^∗∗∗^	-0.19^+^	–	-0.24^∗^	0.10

*Results obtained in Study 1a are shown below the diagonal; results of Study 1b are depicted above the diagonal. ^+^ < 0.07; ^∗^*p* < 0.05, ^∗∗^*p* < 0.01, ^∗∗∗^*p* < 0.001.*

**Table 2:** Regression analyses testing regulatory focus, neuroticism, and extraversion as predictors of generalized trust scores in Studies 1a,b.

Criterion	Predictor	*F*	*R^2^*	*B*	*SE B*	* β*
Generalized trust		7.40^∗∗^	0.148			
	Prevention			-.242	0.073	-0.339^∗∗^
	Promotion			-0.116	0.097	-0.123
Generalized trust		10.83^∗∗∗^	0.279			
	Prevention			-0.443	0.085	-0.574^∗∗∗^
	Promotion			-0.211	0.085	-0.208^∗^
	Neuroticism			0.125	0.088	0.164
	Extraversion			0.065	0.092	0.064

*^∗^p* < 0.05, ^∗∗^*p* < 0.01, ^∗∗∗^*p* < 0.001.

One unexpected aspect of the findings that emerged in Study 1 is the fact that the promotion scale did show a modest negative association with generalized trust scores. One may speculate that this modest negative relation may be attributed to the fact that promotion–focused self-regulation has been proven to be positively related to human values reflecting self-enhancement (achievement and power; cf. Leikas et al., 2009; Keller, unpublished) and negatively related to values reflecting self-transcendence (universalism and benevolence; cf. Leikas et al., 2009; Keller, unpublished). Given that self-enhancement values haven been found to relate negatively to generalized trust (e.g., Tatarko, 2013), the modest negative relation between promotion focus and trust observed in our study may reflect the specific value connotation of promotion-focused self-regulation. However, this is a *post hoc* consideration and as outlined above, there are no strong reasons to predict a negative relation between the promotion focus and generalized trust on conceptual grounds (and in line with this reasoning we did *not* find such a negative relation in the studies reported below).

In combination, the present results support our reasoning that a prevention-focused self-regulatory orientation is inversely related to generalized trust. In the next studies we turn to test our hypothesis within a social interaction that entails threats.

## Study 2

We designed Study 2 to replicate the negative relation between prevention-focused self-regulation and trust using the trust game introduced by Berg et al. (1995). In the trust game, the decision to transfer money to an unfamiliar interaction partner is taken as a good proxy of trustful behavior (e.g., Bohnet and Zeckhauser, 2004; Buchan and Croson, 2004; Kosfeld et al., 2005; Greifeneder et al., 2011). In this experimental game, participants initially receive a certain amount of money. They are informed that they can transfer any amount of this money to a second person and that this amount will be tripled before the other person receives the transfer amount. At this point, the other person (who receives the money) is free to decide whether or not to reciprocate by sending a certain amount of money back. Thus, this paradigm is designed to assess the level of trust on the part of the initiator (i.e., the person who can decide on how much money he or she is willing to put in the second person’s hands). Individuals with a strong tendency to trust are expected to send the full amount to the interaction partner, while the lowest level of trust is represented by the decision not to send any money to the interaction partner. We predict that the more prevention-oriented the person in the position to initiate the money transfer is, the smaller are the chances that he or she is willing to trust another unfamiliar person, in other words – to respond to a threat (of receiving little or no money back) with trust. Accordingly, we expect an inverse, that is, negative, relation between participants’ scores on the measure of prevention-focused self-regulation and the amount of money offered to a (hypothetical) second player.

### Procedure

Seventy-nine students at the University of Mannheim (*M*_age_ = 21.4; 43 women, 32 men; four participants did not indicate their sex) filled out a questionnaire that included a short version of the regulatory focus scale (with three items per subscale; Cronbach’s alpha Promotion: 0.65; Prevention: 0.61) as applied in Study 1 (Lockwood et al., 2002).

To operationalize generalized trust other than by responses to questionnaire items, we relied on a scenario version of the trust game paradigm (cf., e.g., Buchan and Croson, 2004, for uses of the trust game in scenario version). In this scenario version, we asked participants to imagine that they were participating in a study on social behavior and that each participant in this imaginary study initially receives a payment of 12€ for showing up. Moreover, our participants learned that in the imaginary study participants were randomly assigned to the role of either a money transfer initiator, or transfer receiver. They were told that participants in the role of a money transfer initiator would have the option of transferring an amount between 0 and 12€ (the amount of money each participant had received for showing up) to another anonymous person (the receiver) who would then decide on how much money he or she wanted to send back. It was further explained that the amount of money the initiator was willing to transfer (between 0 and 12€) would be *tripled*. That is, if the initiator decided to transfer 4 Euro, the receiver would get 12€ and could then decide on how much of this amount he or she was willing to send back to the initiator. Participants also learned that in the imaginary study the persons involved in the interaction would remain absolutely anonymous and had no chance to communicate with each other.

Following the detailed description of the scenario, participants were asked to imagine themselves in the role of the transfer initiator and to decide on the amount of money they would be willing to transfer to the receiver (on a scale with 13 response options representing units of 1 between 0 and 12€).

### Results and Discussion

**FIGURE 3:**
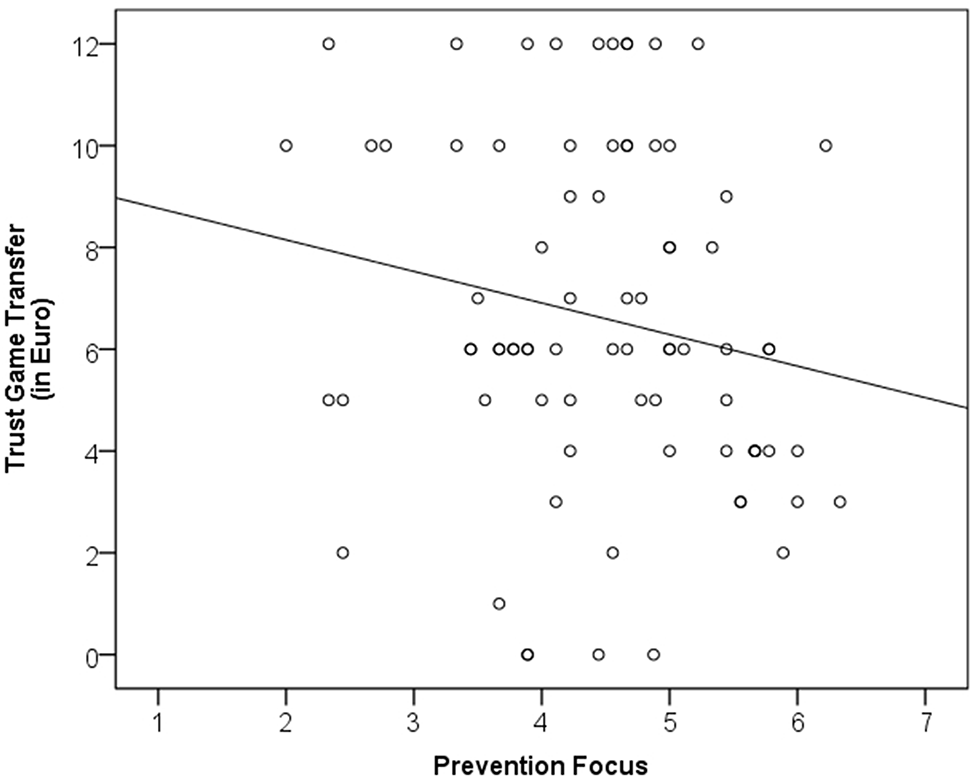
X–Y plot of Study 2.

In line with our theoretical analysis and the findings obtained in Study 1, we expected a negative association between participants’ chronic prevention focus and the amount of money they would be willing to transfer to the interaction partner. Responses in the trust game paradigm varied between 0 and 12€ and the distribution was clearly non-normal (multiple peaks emerged: 17 participants selected 6€, 12 participants selected 10€, and 10 participants selected 12€). Accordingly, we applied non-parametric analyses to assess the relation between regulatory focus scales and trust game responses. We computed non-parametric correlations (Kendall‘s τ) between the Lockwood et al. (2002) focus scales and trust game responses. As expected, the prevention focus was negatively related to generalized trust as reflected in the tendency to give money to a stranger (Kendall‘s τ = -0.18, *p* < 0.03; see **Figure 3**). In contrast, the promotion focus was slightly positively related to trust game responses (Kendall‘s τ = 0.05, *n.s.*) ^3^.

When we entered promotion and prevention scale scores simultaneously in ordered logit regression analyses, we found parallel results. Specifically, prevention scale scores emerged as a significant predictor, Wald χ^2^ = 8.12, *p* < 0.01, the respective odds ratio of 0.61 indicates that a one-unit change in the prevention scale score decreases the odds of the decision to transfer the full amount available (versus the combined other response categories) by 39%. The coefficient for the promotion scale scores was not significant, Wald χ^2^ = 1.07, *p* > 0.30, odds ratio 1.25.

## Study 3

The findings reported thus far consistently support the hypothesis that prevention-focused self-regulation is negatively related to generalized trust. Specifically, prevention-focused self-regulation leads to responding with less trust in a social interaction paradigm that involves a threat. Given that Studies 1 and 2 were based on self-report measures of generalized trust and a scenario version of the trust game, it remains to be tested whether the relation holds when we assess actual trust behavior. To address this critical question, we assessed whether participants’ actual transfer of real money in the trust game paradigm was similarly affected by the individuals’ level of prevention-focused orientation.

### Procedure

Seventy-seven students at the University of Mannheim (*M*_age_ = 22.4; 37 women) participated in this study and received monetary compensation (2€ plus extra money depending on their response in the experimental setting, see description below). Four participants were excluded because they did not follow experimental procedures adequately, leaving a sample size of 73 for subsequent analyses.

When participants arrived at the lab, a confederate of the experimenter appeared and served as ostensible other participant so that participants were made to believe that there was another participant present with whom they would later be interacting. There was no vocal communication between confederate and participants. Participants were told that the other participant would be sitting in an adjacent room and completing the same material. Once seated in their cubicle, participants received a questionnaire that contained the measure of chronic regulatory focus used in Study 1 [Lockwood et al.’s (2002) scales; *α*_Promotion_ = 0.81; *α*_Prevention_ = 0.76].

### Trust Game Paradigm Involving Real Money

After completing the regulatory focus measure, participants received new materials from the experimenter and read that the researchers were interested in individuals’ reactions in social interactions involving financial decision-making. As stated above, participants initially received 2€ (in form of ten 20 Cent coins) for showing up and read in the description of the trust game paradigm that they could exchange this money. Participants learned that they would be randomly assigned either to the role of a money transfer initiator or the role of a transfer receiver (in reality, all participants were assigned to the role of money transfer initiator). Participants in the role of a money transfer initiator had the option to transfer any amount between 0.20 ^4^ and 2€ to another person (the receiver), who would then decide how much money he or she wanted to send back. In line with the general logic of the trust game (as outlined in Study 2 above), it was further explained that the amount of money the initiator was willingto transfer (between 0.20 and 2€) would be *tripled*. Moreover, participants were told that the individuals involved in the interaction would remain strictly separated and had no chance of communicating with each other in the course of the study or after.

Following the detailed description of the scenario, participants were asked to decide on the amount of money they would be willing to transfer to the receiver and to put the respective amount in an envelope provided by the experimenter, who then ostensibly brought the envelope to the transfer receiver in the adjacent room. The experimenter then returned, handed over one of several previously prepared envelopes containing the amount of money that either reflected a repayment of 100% (for participants randomly assigned to the norm violation condition) or a repayment of 160% (for participants randomly assigned to the control condition) and provided the participant with further materials that are not relevant in the present context (i.e., the trust game was part of a different line of research on self-regulation and aggression that has been reported by Keller et al., 2008).

### Results and Discussion

As in Study 2, the distribution of scores representing money transfer decisions was clearly non-normal. Specifically, a large proportion of participants (*n* = 38 or 52.1%) decided to transfer the complete available amount of 2€. This behavior can best be described as a decision to “give all" while the remaining participants decided to “give some" of the available money. Accordingly, we conducted an analysis predicting the decision to “give all" (transfer of 2€) or to “give some" by way of a logistic regression with the respective dummy variable as the criterion. We expected findings replicating the patterns obtained in Study 2, that is, a negative relation between prevention scale scores and the decision to “give all." Results of the logistic regression analysis revealed that prevention scale scores predicted the decision, *B* = -0.52, Wald χ^2^ = 3.68, *p* = 0.055, the respective odds ratio was 0.59. Promotion scale scores were not reliably associated with the trust game decision, *B* = -0.17, Wald χ^2^ = 0.32, *p* = 0.57, odds ratio = 0.83. The same pattern was found using zero-order correlations: analyses revealed that prevention scale scores were significantly negatively related to the decision to “give all" (*r* = -0.25, *p* = 0.03), whereas promotion focus scores were not significantly related (*r* = -0.12, *p* = 0.32).

These results speak to the fact that the inverse relation between prevention-focused self-regulation and generalized trust can be documented not only based on self-report measures of generalized trust and a scenario version of the trust game but also based on actual trust behavior involving real money in the trust game paradigm.

In this context, one could argue that the willingness to transfer money in the trust game paradigm is primarily due to the individuals’ willingness to incur risk. According to this perspective, knowing participants’ tolerance of risk may be sufficient to predict their transfer behavior, regardless of how much or how little they trust the other player. However, such a perspective is incompatible with empirical findings documenting that there are no meaningful relations between measures of risk taking and behavior in the trust game paradigm. For instance, Eckel and Wilson (2004) revealed that neither the Zuckerman (1994) sensation seeking scale (a widely used measure of risk taking) nor behavioral risk measures (involving lottery choices; cf. Holt and Laury, 2002) were related to the decision to trust in the trust game paradigm. Moreover, participants in the trust game seem to care not only about the payoff outcome but “behave as though there is a betrayal cost above and beyond any dollar losses" (Bohnet and Zeckhauser, 2004, p. 474). Kosfeld et al. (2005) showed that risk taking can be differentiated from trust and that risk calculations are of minor relevance in a trust game. Accordingly, it appears fair to conclude that trust game responses do not reflect mere risk taking, but a generalized tendency to trust in the face of a social threat of not receiving something back, as is evident in several investigations documenting substantial positive correlations between trust game responses and self-report measures of trust or reported past trusting behaviors (e.g., Glaeser et al., 2000; Evans and Revelle, 2008). Taken together, this evidence clearly speaks to the validity of the trust game paradigm and is incompatible with the proposition that responses in this paradigm reflect nothing but willingness to take risks. We also want to mention that the findings including the trust game are complemented by measures assessing individual differences in trust (Studies 1 and 2) where risk calculations are less likely to be relevant. Here, the same pattern emerged, that is, prevention focus is negatively related to individual differences in trust.

## Study 4

Studies 1–3 demonstrate that chronic prevention focus is inversely related to generalized trust. In a next step we extended these finding and tested whether a parallel relation of prevention-focused self-regulation and generalized trust can be documented when prevention focus is situationally activated. It is evident that this would be an important contribution to the current line of research in that we could draw firmer conclusions regarding the (causal) nature of the relation in question. Accordingly, we conducted an experimental study involving the manipulation of the prevention focus and we assessed participants’ responses in the trust game paradigm (parallel to Study 2) following exposure to this manipulation.

### Procedure

Sixty students at the University of Mannheim (*M*_age_ = 22.0; 30 women) participated in this study. They were randomly assigned to one of two experimental conditions (see below) and received a questionnaire containing the relevant materials.

### Prevention Focus Manipulation

In order to induce a prevention focus, half of the participants were asked to report on their duties and obligations (following the logic proposed by Higgins et al., 1994; see also Freitas et al., 2002). Specifically, we provided half of the participants with a description of the concept of the ought self followed by a statement referring to the fact that a defining feature of the ought self was the fear of (a) not possessing the respective elements of the ought self and (b) being punished or rejected because of an existing discrepancy between the actual and ought self. Following this introduction, participants noted three traits or characteristics that reflected elements of their ought self. Finally, we asked participants to indicate the person(s) that considered the indicated elements of their ought self as relevant. That is, participants mentioned the person(s) who made them feel responsible (or obligated) to possess the respective trait or characteristic and who may punish or reject them in case of an existing actual-ought discrepancy. The other participants (in the control condition) did not receive this part of the questionnaire.

As a measure of generalized trust, we used again a scenario version of the experimental trust game paradigm (parallel to Study 2; see also Buchan and Croson, 2004). That is, the main dependent variable in this study was the amount of money participants were willing to transfer to the receiver (on a scale with 13 response options representing values between 0 and 12€).

### Results and Discussion

In line with our theoretical analysis and the findings obtained in Studies 1–3, we expected a lesser amount of money to be transferred in the condition of prevention focus induction compared to the control condition. Responses in the trust game paradigm varied between 0 and 12€ and the distribution was again clearly non-normal (multiple peaks emerged: nine participants selected 6€, and 17 participants selected 12€). Accordingly, we applied a non-parametric test to assess the effect of regulatory focus priming on the amount of money transferred. A Mann–Whitney-*U*-Test revealed that participants in the prevention prime condition offered significantly less money to their ostensible interaction partner (*Median* = 5.0) than their control group counterparts (*Median* = 8.5, *z* = 1.97, *p* < 0.05). The results of Study 4 thus support the proposition that prevention focus decreases the expression of generalized trust. This finding–which is based on an experimental manipulation of the prevention focus–speaks to the causal nature of the relation between vigilant prevention-focused self-regulation and generalized trust as well as to the fact that situational variations (in extension to chronic differences) in prevention focus affect generalized trust.

Interestingly, alternatively to what we hypothesized, one could argue that the experimental group differed from the control group not only in the activation of a prevention focus, but also with regard to other factors such as negative mood or self-awareness. To address such alternative hypotheses, we assessed potential effects of the experimental procedure (as applied in the current study) on participants’ mood state in a separate pre-test (*n* = 39). The item used to assess participants’ mood state reads “How do you feel right now?" with response scale end poles labeled (1) *in a good temper* and (9) *in a bad temper*. We did not observe a meaningful effect, *F*(1,37) = 0.002, *p* > 0.96. Accordingly, an alternative interpretation referring to mood seems not particularly likely. Note that the possibility that the procedure applied in Study 4 may have increased experimental participants’ self-awareness or general self-focus will be addressed in Study 5 where we made use of a different procedure to induce a prevention focus that is unlikely to trigger a self-focus or self-awareness.

## Study 5

Study 4 complemented the correlational findings observed in Studies 1–3 by situationally inducing a prevention-focused self-regulatory orientation. Although intriguing and consistent, this evidence is silent about the specific mechanisms involved in the relation. Study 5 therefore relies on an experimental paradigm designed to test the specific impact of two crucial elements proposed as characteristic aspects of prevention-focused self-regulation: sensitivity to negative information and the need for security.

In this study, we made use of a fairly subtle ‘problem cue’ referring to the need for security that is known to be related to prevention-focused self-regulation (Friedman and Förster, 2001). This cue should decrease individuals’ trust in another person. Hence, ceteris paribus, the provision of a prevention-triggering cue should result in lower levels of trust. Moreover, we assessed habitual differences in the sensitivity to negative information as a second crucial factor. Given our previous results, one may suppose that both factors – sensitivity to negative information and the need for security – affect individuals’ tendency to trust strangers (which would be reflected in main effects of both factors). However, as an alternative one may expect an interplay of both factors such that the fairly subtle problem cue (referring to the need for security) is only effective in individuals with a strong sensitivity to negative information. In our view, such an interplay seems likely to occur given the theoretical and empirical background as discussed in the next section.

### Differential Sensitivity to Problem Cues Signaling Potential Negative Events

As outlined in the description of the proposed consequences associated with each style of self-regulation, RFT holds that individuals are sensitive to different outcomes and consequences depending on the mode of self-regulation that is guiding their regulatory system (Higgins, 1997, 1998). Specifically, RFT predicts that the prevention focus is related to a special sensitivity concerning negative outcomes and consequences, whereas the promotion focus is related to a special sensitivity concerning positive outcomes and consequences. Starting from this proposition, we suggest (based on previous evidence; Keller et al., 2008; cf. Keller, unpublished) that a sensitivity to negative outcomes and consequences involves a specific vigilance regarding environmental cues that signal insecurity and potential losses (i.e., signals indicating that there is a potential for negative consequences in the current situation). And we propose that this special sensitivity to cues that signal insecurity and potential losses can be related to the inverse relation between prevention-focused self-regulation and generalized trust (as observed in the previous studies).

In fact, it appears quite adaptive to lower one’s willingness to trust another person in situations where one perceives situational cues indicating potential negative consequences. However, individuals differ in how quick they are in detecting and interpreting cues as signals of insecurity and potential losses – and the style of self-regulation is most likely associated with such individual differences. We obtained empirical evidence in support of this notion (Keller, unpublished) in two independent correlational studies, which yielded significant positive correlations between prevention (but not promotion) focus scale scores and two instruments designed to assess individual differences in the sensitivity to negative information as reflected in (a) a cognitive tendency to focus on negative information (Noguchi et al., 2006) and (b) a tendency to follow negative emotions (Gasper and Bramesfeld, 2006).

Building on these previous findings documenting a close association between prevention-focused self-regulation and sensitivity to negative information, we assume that individuals high on prevention-focused self-regulation have a strong chronic sensitivity to negative information and therefore are particularly vigilant with respect to subtle cues signaling insecurity, and a potential for negative consequences. Accordingly, we argue that confrontation with a subtle prevention focus problem cue (a subtle trigger of the need for security) is most likely to result in defensiveness and a decreased willingness to express trust among individuals characterized by a special sensitivity to negative information or cues in the environment.

To test these considerations, we conducted an experimental study involving the manipulation of a subtle prevention focus problem cue and we assessed participants’ sensitivity to negative information as a critical boundary condition.

### Procedure

Forty students at the University of Mannheim (*M*_age_ = 25.6; 20 women) participated in this study and received 1€ and a chocolate bar as compensation. They were randomly assigned to one of two experimental conditions. All materials were combined in one questionnaire, including the manipulation of the prevention focus problem cue, the scenario version of the trust game paradigm, and a personality measure of sensitivity to negative information ^5^.

### Manipulation of Prevention Focus Problem Cue

We applied a procedure developed by Friedman and Förster (2001) as a subtle prevention focus priming task. In this task, participants complete a simple paper-and-pencil maze that is designed in a way that there is a cartoon mouse depicted trapped inside the maze and participants are instructed to “find the way for the mouse." Outside the maze, a brick wall is depicted containing a mouse hole. In the prevention focus version of the maze task, an owl is depicted as hovering above the maze presumably ready to fly down and capture the mouse unless it could escape the maze and retreat through the mouse hole. This manipulation reflects a subtle activation of the need for security. Participants in the control group condition received a questionnaire that did not contain this maze task.

### Trust Game Paradigm

The description and procedure of the trust game scenario was parallel to that applied in Studies 2 and 4.

### Sensitivity to Negative Information

We assessed participants’ sensitivity to negative information with a measure comprising 10 items of the “attention to negative information scale" developed by Noguchi et al. (2006; a sample item reads “I notice when something is not going well even if it’s a trivial thing") as well as the four items taken from Gasper and Bramesfeld’ (2006) “following negative emotions" subscale of the “Following Affective States Test" (one sample item reads “I do pay attention to my negative feelings"). The combined 14-item scale was internally consistent with Cronbach’s α = 0.86.

### Results and Discussion

In parallel to Studies 2–4, participants’ trust game responses were non-normally distributed which is why we conducted non-parametric tests. In an analysis using ordered logit regression we observed no significant main effects whereas a significant interaction effect involving sensitivity to negative information and the problem cue dummy variable emerged, Wald χ^2^ = 0.4.42, *p* < 0.04. In additional focused analyses (cf. Aiken and West, 1991; Hayes, 2013) we found that the prevention focus problem cue manipulation had the expected significant effect. Specifically, participants scoring relatively high on the sensitivity to negative information scale (i.e., 1 *SD* above mean) who were confronted with the prevention focus problem cue decided to transfer significantly less money to the ostensible other player as compared to the control condition (*B* = -2.38, *t* = -2.82, *p <*0.01). In contrast, the prevention focus problem cue manipulation had no significant effect among participants scoring relatively low (i.e., 1 *SD* below mean) on the sensitivity to negative information scale (*B* = 0.26, *t* = 0.30, *p* = 0.76). In sum, the findings obtained in this experimental study suggest that the special sensitivity to negative information that is characteristic of prevention-focused self-regulation is associated with a strong tendency to react with distrust when confronted with a social threat signaling danger and insecurity. This supports the RFT perspective according to which prevention-focused individuals’ defensiveness is associated with a particular sensitivity to environmental cues that signal insecurity and a potential for negative consequences. Moreover, the obtained results support the notion that trust game responses vary as a function of situationally induced prevention focus mechanisms. This indicates – parallel to Study 4 – that there is indeed a causal relation between prevention focus mechanisms and generalized trust. This finding complements and extends the correlational results obtained in the Studies 1–3.

In Study 5, we did not observe a main effect of the prevention focus problem cue manipulation. In our view, this is most likely due to the fact that we used a rather *subtle* method to elicit prevention-focused-concerns to which only individuals with a strong sensitivity to negative information are responsive.

## General Discussion

In relating dis-/trust to RFT (Higgins, 1998, 2012a,b), we propose that a prevention-focused mode of self-regulation is inversely related to generalized trust. The obtained findings support this hypothesis. Across a series of studies, participants consistently scored lower on measures of generalized trust the more they were prevention-focused in their self-regulatory orientation. In these studies, we applied different measures to assess individual differences in regulatory focus, we also situationally manipulated prevention-focused mode of self-regulation and tested the effect regarding different measures of generalized trust. The proposed relation was observed irrespective of the type of measurement applied and for both prevention-focused orientation as a disposition as well as a situational factor. Accordingly, the obtained evidence can be considered strong support for the proposed inverse relation between prevention-focused self-regulation and generalized trust.

Going beyond the ‘first generation of research’ (Zanna and Fazio, 1982) that looked at the question of whether a relation between (prevention-focused) self-regulatory mechanisms and generalized trust can be observed, we also addressed the second-generation question focusing on the specific factors involved in the observed relation (see also Fiedler and Krueger, 2013). Specifically, we tested differential sensitivity to negative information and the need for security as relevant factors and found empirical support for the notion that these specific factors – in combination – play a crucial role and contribute to substantial differences in the tendency to trust unfamiliar others.

The reported research offers several innovative insights. First, in relating RFT to generalized trust we document the crucial impact of self-regulatory mechanisms with regard to a very important social interactive phenomenon that reflects a fundamental basis of social life. Trust is an important factor in interpersonal relationships, intergroup relations, as well as in social organizations. Accordingly, understanding the mechanisms involved in the emergence of trust (or distrust) among unfamiliar persons is extremely relevant, and our work contributes to this field of research by providing insights from a self-regulatory perspective. The role of self-regulatory mechanisms has not been assessed previously in the analysis of generalized trust, and the present contribution therefore represents a new and promising approach. Our analysis reveals the relevance of vigilant self-regulatory mechanisms regarding individuals’ behavior in important interpersonal situations.

Second, we put the differential sensitivity assumption entailed in RFT to a critical test in the context of trust and observed strong support. Specifically, we found that participants with a strong sensitivity to negative information were particularly sensitive with respect to a subtle prevention focus problem cue. The findings obtained in Study 5 suggest that prevention-focused individuals are particularly likely to react defensively, specifically with distrust, when confronted with a subtle sign related to danger and insecurity. This particular aspect of prevention-focused self-regulation has not been documented before and thus represents an extension of our knowledge on the specific mechanisms characteristic of this distinct self-regulatory mode.

In sum, the current findings can be viewed as a contribution to the understanding of the role self-regulatory mechanisms play concerning generalized trust, and they enrich our knowledge about the factors that contribute to the expression of trust (or distrust) among unfamiliar persons. As such, the current studies open a new avenue of research for studying trust that incorporates the crucial impact of self-regulatory mechanisms.

## Avenues for Future Research

We want to note that the analysis of self-regulatory mechanisms with respect to interpersonal trust in the domain of (close) ongoing relationships may result in different predictions and results than those discussed in the present contribution focusing on generalized trust. Specifically, one could speculate that in the context of close relationships individuals with a strong prevention focus could be particularly motivated to override their fears of becoming vulnerable and to trust the persons in their intimate social environment in order to attain security that a trusted other can provide. That is, the desire for security may be satisfied via the establishment of trusting close relationships and this strategy may be particularly attractive to prevention-focused individuals. Consequently, the conclusions offered in the present contribution may not necessarily translate to interpersonal trust in (close) ongoing relationships. Of course, the fact that we prefer to remain silent with respect to the domain of (close) relationships does not imply that we consider self-regulatory mechanisms as irrelevant or unimportant in the domain of ongoing partnerships or close relationships. To the contrary, it appears to be an intriguing topic on the agenda of research on self-regulation and trust that should be addressed in future research.

One could also discuss boundary conditions of the observed relation between prevention-focused self-regulation and generalized trust. Scholer et al. (2010) show that a prevention focus results in increased risk taking under the conditions of a loss. In the present studies, individuals are not in the state of a loss because they have received a specific amount of money to make their trust decisions. However, on basis of the work by Scholer et al. (2010) one could argue that prevention-focused individuals behave differently in a state of a loss by increased trust. Indeed, the possible distinct effect of dealing with a *potential* loss, as considered in the current studies, compared to dealing with a situation of a factual loss as considered by Scholer et al. (2010) is an interesting aspect for future research to address.

## Implications

Given the strong relevance of generalized trust in everyday life, we would like to end with a discussion of several practical implications of the present work with respect to potential means for increasing individuals’ tendency to trust others. In the most general terms, our finding of an inverse relation between prevention-focused self-regulation and trust suggests that individuals’ tendency to trust may be enhanced by reducing the strength or relevance of needs and motives that are related to prevention-focused self-regulation. That is, it seems plausible to assume that individuals are more likely to express trust and behave in a trusting way when the needs and motives underlying prevention-focused self-regulation have been satisfied and are therefore not likely to control and govern the self-regulatory orientation in the respective situation. A related important factor is the salience of losses and potential negative outcomes in the situation and hence the accessibility of related thoughts and the activation of a pessimistic mindset. Our findings suggest that trust is more likely to emerge the less individuals are guided by the desire to avoid losses or negative outcomes. Thus, situational cues and inputs that trigger an optimistic perspective (and decrease the accessibility of pessimistic and misanthropic thoughts and related motives) are likely to contribute to an increased tendency to express trust in people.

Another possible strategy to counter prevention-focused individuals’ tendency to distrust could be to make use of prevention-focused individuals’ respect for normative standards and to emphasize that trusting behavior is normatively appropriate. Based on the notion that prevention-focused individuals are particularly concerned with fulfillment of oughts and responsibilities, such a strategy seems, in theory, particularly meaningful.

In sum, the present work builds the basis for a systematic analysis of factors related to basic self-regulatory mechanisms that contribute to the expression of trust (or distrust) and it has the potential to contribute substantially to our understanding of possible strategies to foster the development and expression of generalized trust. Given that generalized trust is declining in the general population (cf. Paxton, 1999; Robinson and Jackson, 2001), and given that generalized trust is important for organizations and democratic systems to function appropriately (cf. Almond and Verba, 1963; Uslaner, 1999), this seems to be an issue of great relevance.

## Conflict of Interest Statement

The authors declare that the research was conducted in the absence of any commercial or financial relationships that could be construed as a potential conflict of interest.

## Footnotes

1 We are well aware of the fact that following the false positive debate it is now considered desirable to conduct studies with a determination of sample sizes based on power analyses. Please note that the studies reported in the current manuscript were conducted well before the false positive debate was initiated in 2011. Accordingly, we are not in a position to refer to power analyses that were conducted prior to the data collection. What we did was that we took into consideration previous studies in the field of regulatory focus research regarding correlations between measures of individual differences in regulatory foci and measures of other trait level constructs. These considerations suggested that sample sizes between 80 and 120 typically provided enough power to detect meaningful associations.

2 We also assessed an alternative regulatory focus instrument developed in our lab (Keller, unpublished) that has been used in several studies (cf. Keller and Bless, 2008; Leder et al., 2013, 2015). The alternative regulatory focus scale was assessed after the Lockwood scale in this study (with six items taken from the need for cognition scale as filler items between the Lockwood scale and the alternative regulatory focus scale). A sample promotion-item of this instrument reads: “If I know that my performance is being evaluated by other people that spurs me on and increases my ambition to do well." A sample prevention-item reads: “My life is often shaped by fear of failure and negative events." Both scales were internally consistent and reached acceptable Cronbach’s alpha levels (Promotion: 0.65; Prevention: 0.81). Note that these scales are conceptually similar to the Lockwood et al. (2002) scales (cf. Keller, unpublished). For reasons of brevity, and given that the Lockwood et al. (2002) instrument is the more established measure of chronic regulatory focus, we focus on these latter scales in the results section.

3 Parallel results emerged in this study (as well as in the studies reported below) when we conducted parametric analyses (i.e., “classic" regression analyses). Also, the observed pattern of results was corroborated when analyzing the relation between trust and prevention focus as measured with Keller’s scale. This scale was assessed directly preceding the short version of Lockwood scale. Results based on Keller’s (unpublished) scale showed a similar picture. The prevention scale revealed a negative relation (Kendall‘s τ = -.16, *p* < 0.06); the promotion scale was marginally significantly positively related with trust game responses (Kendall‘s τ = 0.15, *p* > 0.07).

4 In this particular study, we offered no option to refrain from transferring money (0[€]) because the trust game paradigm was used in the context of another research project in order to manipulate perceived fairness in terms of the back transfer of money by the ostensible interaction partner (cf. Keller et al., 2008); accordingly, the 0[€] response option was omitted in this version of the trust game paradigm to ensure that a money exchange was actually involved in every session of the study.

5 We assessed sensitivity to negative information after the manipulation and the assessment of trust game responses in order to avoid possible negative sensitivity priming effects that may have emerged if we had asked participants to respond to the items of the scale prior to the manipulation. Given that the measure has been designed as an instrument to assess a stable disposition it seems unlikely that the preceding procedures could affect participants’ scores on this scale. Supporting this assumption, we found that the problem cue prime had no impact on participants’ sensitivity to negative information scores, *F* < 0.2. Note that a similar posttest measurement strategy has been used successfully in several influential social psychological studies (cf. Devine and Elliot, 1995; Lepore and Brown, 1997; Wittenbrink et al., 1997).
